# Experimental Study on Seismic Behavior of Concrete-Filled Steel Tube with Spherical-Cap Gap

**DOI:** 10.3390/ma17225538

**Published:** 2024-11-13

**Authors:** Aimin Xu, Dewei Liu, Jiuhong Fan, Jin Di, Jie Wang, Fengjiang Qin

**Affiliations:** 1 Ningbo High-Grade Highway Construction Management Center, Ningbo 315192, China; 13335746588@139.com; 2 Sichuan Shudao New System Rail Group Co., Ltd., Chengdu 610047, China; qiliang_1208@126.com; 3 Key Laboratory of New Technology for Construction of Cities in Mountain Area, School of Civil Engineering, Chongqing University, Chongqing 400030, China; dijin@cqu.edu.cn (J.D.); wangjie205421@163.com (J.W.)

**Keywords:** concrete-filled steel tube (CFST), spherical-cap gap, quasi-static test, seismic behavior

## Abstract

Concrete-filled steel tubes (CFST) are widely used due to their high strength, ductility, and energy dissipation capacity. However, gaps in between core concrete and steel tube adversely affect the mechanical performance of structures, thereby compromising the safety of the building. In this paper, four concrete-filled steel tube specimens with spherical-cap gaps were designed, and quasi-static tests were conducted to investigate the impact of gap depth on the seismic performance of concrete-filled steel tube columns. The test results indicate that the gap reduced the cumulative energy dissipation and initial stiffness of concrete-filled steel tubes. The gap weakened the compressing effect on the steel tube exerted by the expansion of core concrete, leading to premature yielding of the steel tube. As the gap’s depth increased from 0 mm to 30 mm, the load-bearing capacity and ductility of the concrete-filled steel tube columns decreased by 24.86% and 21.7%, respectively. This research quantified the extent to which gaps weaken the seismic performance of CFST columns, and the reduction coefficients of bearing capacity under different gap ratios were provided. This contributes to enhancing structural safety and lays a foundation for further research.

## 1. Introduction

A concrete-filled steel tube (CFST) combines concrete and a steel tube, maximizing the advantages of concrete’s compressive strength and steel’s ductility [1,2,3,4,5]. CFST columns are widely utilized in structures, particularly in high-rise buildings, bridges, and seismic-resistant structures, due to their superior load-bearing capacity, improved seismic performance, and efficient construction process [6,7,8].

The hysteresis performance of CFST columns is a critical factor in determining their reliability during seismic events. High-hysteresis performance indicates a structure’s capacity to absorb and dissipate energy, thereby reducing the likelihood of catastrophic failure during earthquakes [9,10,11]. However, in practical engineering, gaps may exist between the steel tube and the core concrete due to factors such as construction tolerances, material shrinkage, thermal expansion, and inadequate compaction of concrete pouring. These gaps can alter the composite action between steel and concrete, affecting the overall mechanical behavior of CFST columns. Related research on the effect of gaps on the mechanical behavior of concrete-filled steel tubes has been reported.

Liang et al. [12] conducted axial compression tests, eccentric compression tests, and bending tests on CFST members to investigate the effects of gap depths on their performance. The results indicated that gap defects cause local premature yielding of the steel tube, thereby reducing the axial compressive strength. However, the gaps have little impact on the bending and eccentric compressive strengths of the members. Research by Liu et al. [13,14,15] indicates that a higher gap ratio and eccentricity ratio result in a lower ultimate bearing capacity, while the gap has little impact on the ductility of the members. The study of Liu [16] shows that the gap has no effect on the elastic stiffness of single circular tube arch ribs but reduces the elastic stage stiffness of dumbbell-shaped and multi-limb lattice section arch ribs. The bearing capacity of voided multi-limb lattice section arch ribs decreases with an increase in the arch rib eccentricity and slenderness ratio and increases with a higher steel ratio. Numerical analysis by Du et al. [17] indicates that the compressive load on the gap side of CFST columns has a more adverse effect on the bearing capacity compared to the non-gap side. For members with larger slenderness ratios, the impact of the gap ratio on the bearing capacity is even more pronounced. The research of Ye [18] indicates that when the gap ratio is below 0.1%, the bearing capacity of square CFST short columns decreases by less than 5%. However, when the gap ratio exceeds 0.4%, the steel tube and the core concrete bear loads independently before the failure of the member, resulting in a reduction of approximately 20% in the bearing capacity.

Zhang et al. [19] and Zhang et al. [20] conducted cyclic loading tests on CFST under constant axial compression and repeated bending–torsion coupling loads, investigating the effects of gap ratio, axial compression ratio, and bending–torsion ratio on the hysteresis behavior of CFST. The results indicated that gap defects have little effect on the shape of the hysteresis curve of CFST but lead to a slight reduction in the stiffness of the post-peak strengthening phase. As the gap ratio increases, the bearing capacity, stiffness, and energy dissipation capacity of the specimens decrease. Liao et al. [21,22] conducted quasi-static tests to study the impact of circumferential gap defects on the seismic performance of CFST specimens under combined compressive, bending, and torsional loads. The results indicate that circumferential gap defects alter the failure mode of the CFST and cause a reduction in their bearing capacity, stiffness, and energy dissipation capacity. A calculation method for the bearing capacity reduction factor, considering the effects of gap, was proposed, providing a reference for practical engineering design and safety assessment. Cyclic loading tests were conducted by Yang et al. [23] on T-joints with spherical-cap gaps. The results indicated that due to insufficient support of the chord wall by the core concrete, local buckling and welding tears near the joint area occurred earlier than in gap-free specimens. The presence of gaps led to greater stiffness degradation and reduced energy dissipation capacity, though the ductility of the joints was generally not significantly affected.

While there have been numerous studies on the mechanical behavior of concrete-filled steel tube columns with gap defects under axial and eccentric loads, research on their seismic performance impact is relatively scarce. Concrete-filled steel tubes are generally recognized for their strong energy dissipation capability and ductility. However, the presence of gap defects may lead to a decrease in seismic performance, which is detrimental to both structural integrity and human safety. Therefore, investigating the influence of gap defects on the seismic performance of concrete-filled steel tube columns is highly necessary.

In this paper, quasi-static tests on CFST columns with a spherical-cap gap were conducted, investigating the effects of gap ratios on load-bearing capacity, ductility, energy dissipation capability, and stiffness degradation of CFST columns. The steel tube and concrete in the specimen experience gaps only in a certain height at the lower part of the column, rather than along its entire length. This study quantified the extent to which gaps weaken the seismic performance of CFST columns and provided the reduction coefficients of bearing capacity under different gap ratios. This study preliminarily investigates the seismic behavior of CFST columns with spherical-cap gaps, aiding designers in understanding their impact.

## 2. Quasi-Static Test

### 2.1. Specimen Details

**Figure 1:**
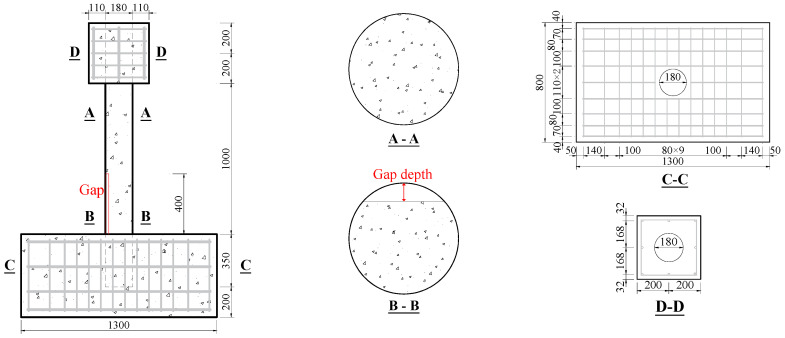
Dimensions of specimens (unit: mm).

Four CFST specimens with spherical-cap gaps (including the reference one) were designed for quasi-static tests. The concrete used for the specimens was C50 in standard GB 50010 [24], and the steel tube was Q345 in standard GB 50017 [25]. The spherical-cap gaps were located within the 400 mm height above the upper surface of the base, as shown in Figure 1. According to code GB 50936 [26], the embedment depth of CFST should not be lower than 1.5 times the column diameter. In this research, a depth of 350 mm (greater than 1.5 × 180 = 270 mm) was conservatively chosen to ensure reliable connection during the test. The dimensions of the upper loading block are 400 mm × 400 mm × 400 mm, and the dimensions of the reinforced concrete base are 1400 mm × 800 mm × 550 mm. The diameter of longitudinal reinforcements in footing and upper loading block is 12 mm, and the diameter of stirrups in footing and upper loading block is 6 mm. The grade of the longitudinal reinforcements and stirrups are HRB400 [24].

The specimen parameters are shown in Table 1. Specimen naming follows the “G-D” principle, where “G” represents the gap and “D” represents the gap’s depth, which are 0 mm, 10 mm, 20 mm, and 30 mm, respectively. The gap ratio represents the ratio of the gap area to the total cross-sectional area of the CFST, and the specimen height is the length from the top of the reinforced concrete base to the loading point.

**Figure 2:**
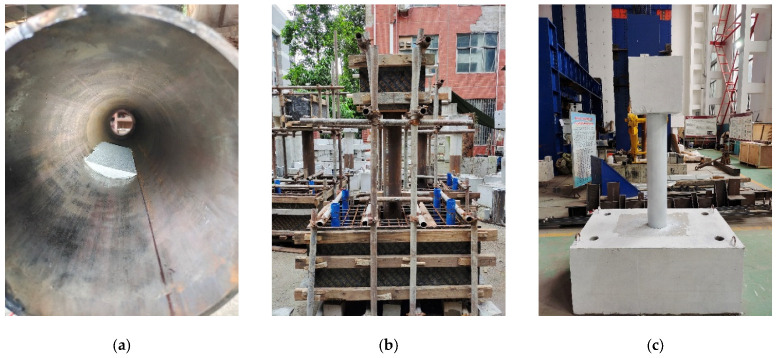
Production of the specimens. (**a**) Affix the foam; (**b**) formwork and casting; (**c**) spray paint.

Before pouring the concrete, affix the prefabricated foam to the designated positions on the steel tube, as shown in Figure 2. Place the steel tube in the predetermined position within the reinforced concrete base formwork. After installing and securing the formwork, pour the concrete and cure it for 28 days. Once cured, grind the rust from the surface of the steel tube and spray it with white paint to facilitate experimental observation.

### 2.2. Material Properties

**Figure 3:**
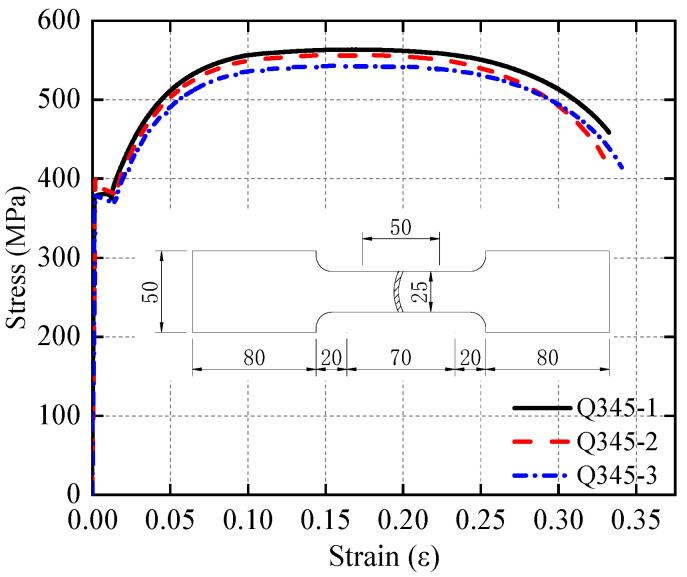
Tensile test result of steel coupon (unit: mm).

Three cubic coupons with dimensions of 150 mm × 150 mm × 150 mm and six prismatic coupons with dimensions of 150 mm × 150 mm × 300 mm were cast using the same batch of concrete as the specimens. After curing the concrete specimens for 28 days, the strength and elastic modulus were tested using a press with a maximum load capacity of 2000 kN. The cubic coupons test followed the provisions of the standard GB/T 50081 [27]. The obtained concrete properties are listed in Table 2. For the steel tube, tensile coupons were cut from 6 mm thick Q345 steel plates according to standard GB/T 228.1 [28]. The dimensions of tensile coupons and stress–strain curves obtained from the test are illustrated in Figure 3, and the material properties are listed in Table 3.

### 2.3. Test Setup and Measurement Layout

**Figure 4:**
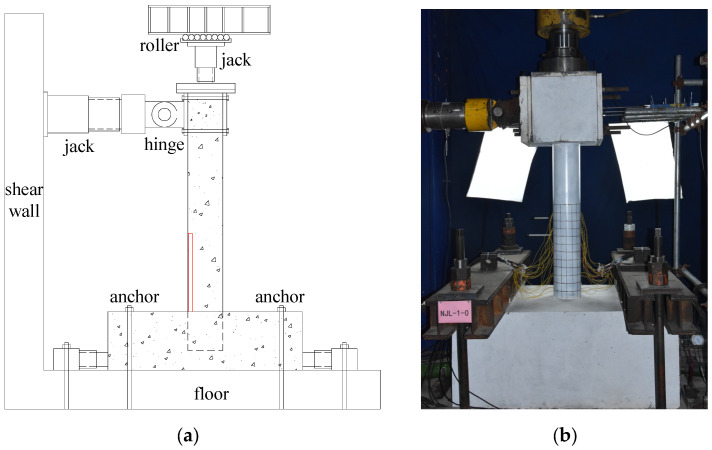
Loading device for quasi-static test. (**a**) Test setup; (**b**) test site.

The quasi-static test for the CFST specimens with spherical-cap gaps adopted a cantilever column loading method, as shown in Figure 4. The reinforced concrete base was anchored to the ground and fixed on both sides with jacks to prevent lateral movement. The axial force was applied to the top of the column via a vertical jack, and a force sensor with a spherical hinge was set between the vertical jack and the specimen, ensuring that the axial force remained vertical during the loading process. To allow the vertical jack to move laterally with the top of the column, rollers were installed at its base and connected to the reaction frame. The horizontal load was applied by a lateral push–pull jack, which was anchored to the shear wall.

**Figure 5:**
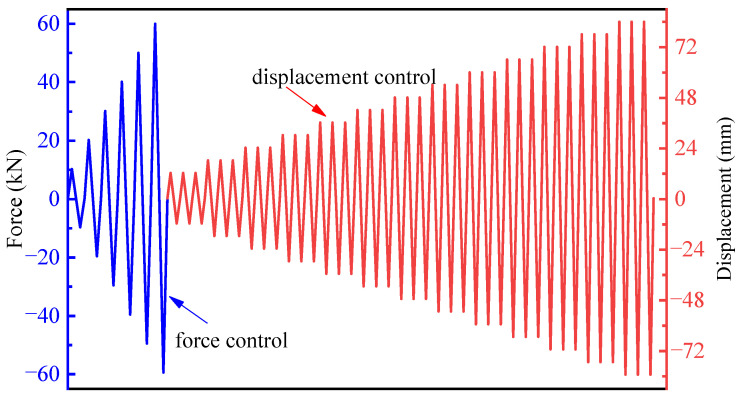
Scheme of lateral load.

Initially, the vertical axial force was loaded to 300 kN, with an axial compression ratio of 0.15, and kept constant during the loading process. Next, the lateral loading was conducted using a force–displacement hybrid control method, as shown in Figure 5. The lateral load is defined as positive for push and negative for pull. The lateral load was first applied in increments of 10 kN, with one cycle per level, until it reached 60 kN, which was the yield load obtained by finite element analysis before the test. Subsequently, a displacement increment of 6 mm, corresponding to a 0.5% drift ratio, was applied, with three cycles at each level. In references [29,30], a 1% drift ratio was used as the increment during the later stages of loading. In this paper, a 0.5% drift angle was used as the increment to better observe the damage process of the specimens. Loading continued until the lateral force dropped to 85% of the peak lateral force or below.

**Figure 6:**
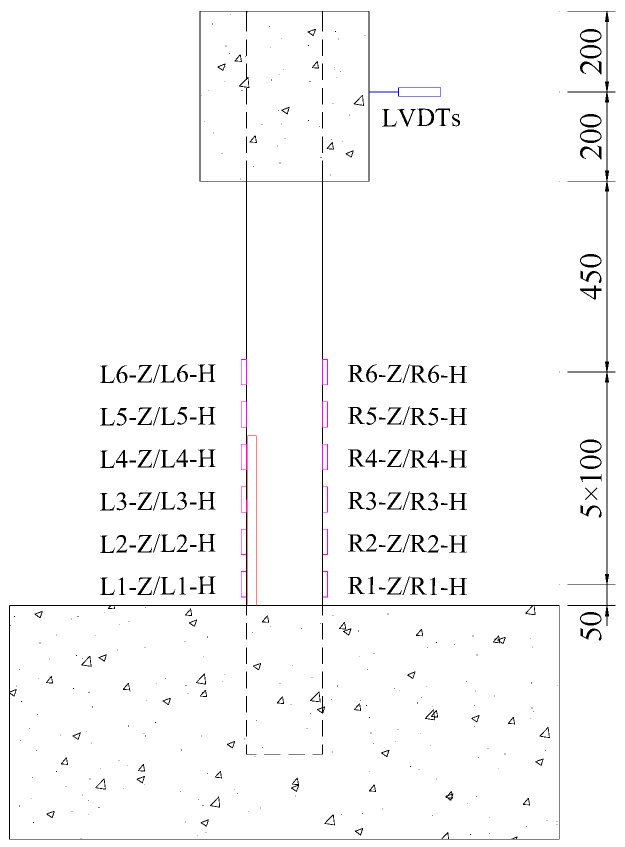
Measurement arrangement (unit: mm, the red represents the gap, the magenta represents the strain gauge).

The arrangement of displacement and strain measurements is shown in Figure 6. Three displacement gauges (LVDT) were placed laterally at the mid-height of the loading block to measure the lateral displacement during the loading process. At the section 50 mm above the top surface of the base and every subsequent 100 mm section, one longitudinal strain gauge and one circumferential strain gauge were placed on both opposite sides of the steel tube. In total, 24 strain gauges were arranged in six rows.

## 3. Test Results and Discussion

### 3.1. Observations and Failure Modes

**Figure 7:**
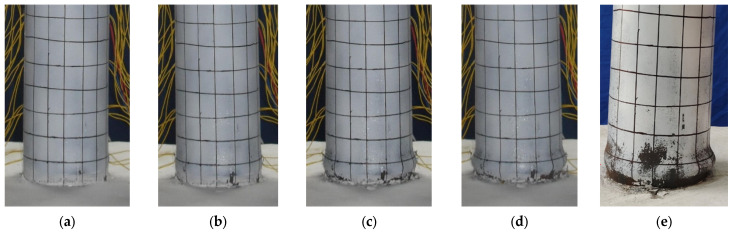
Damage development of specimen G-0. (**a**) ∆ = 36 mm; (**b**) ∆ = 48 mm; (**c**) ∆ = 60 mm; (**d**) ∆ = 84 mm; (**e**) final failure.

The damage development of specimen G-0 is shown in Figure 7. Slight separation between the bottom of the steel tube and the reinforced concrete base was observed when the horizontal load reached 30 kN. When the lateral displacement (∆) reached 36 mm, the load increased slowly, almost approaching its peak. At this point, slight bulging occurred within 50 mm above the base. During the second cycle at 48 mm, the load-bearing capacity decreased by approximately 5 kN. When the displacement reached 60 mm, the bottom of the steel tube exhibited noticeable bulging, taking on a “lantern” shape. Finally, during the third cycle at 84 mm displacement, the load-bearing capacity dropped to 65% of the peak load, and the specimen showed significant inclination.

**Figure 8:**
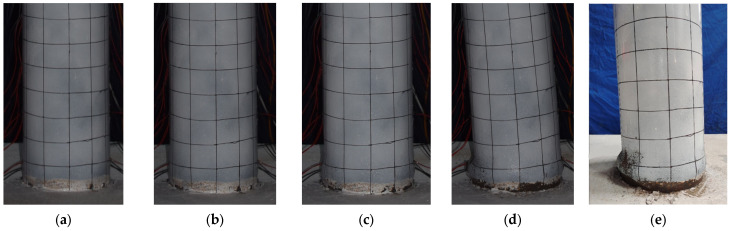
Damage development of specimen G-10. (**a**) ∆ = 36 mm; (**b**) ∆ = 48 mm; (**c**) ∆ = 60 mm; (**d**) ∆ = 84 mm; (**e**) final failure.

The damage development of specimen G-10 is shown in Figure 8. When the lateral load reached 30 kN, a slight separation between the bottom of the steel tube and the reinforced concrete base was observed. When the displacement reached 36 mm, the load increased slowly, almost approaching its peak, with slight bulging at the bottom of the steel tube. At a displacement of −48 mm in the first cycle, slight inward buckling of the steel tube was observed in the left-gapped region. During the third cycle at a displacement of 60 mm, the bottom of the steel tube exhibited noticeable bulging, taking on a “lantern” shape. At a displacement of 72 mm in the first cycle, the bearing capacity decreased significantly. Finally, during the first cycle at a displacement of 84 mm, the specimen showed significant inclination, the horizontal load dropped sharply, and the test was stopped.

**Figure 9:**
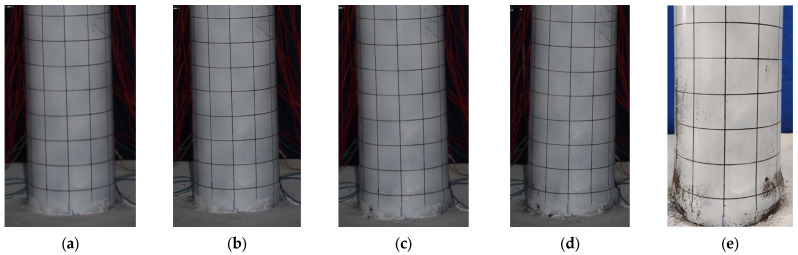
Damage development of specimen G-20. (**a**) ∆ = 36 mm; (**b**) ∆ = 48 mm; (**c**) ∆ = 60 mm; (**d**) ∆ = 72 mm; (**e**) final failure.

The damage development of specimen G-20 is shown in Figure 9. When the horizontal load reached 30 kN, slight separation between the bottom of the steel tube and the reinforced concrete base was observed. During the third cycle at 36 mm, the load increased slowly, and slight bulging occurred at the bottom of the steel tube. At −48 mm in the second cycle, slight inward buckling of the steel tube was observed in the left-gapped region. During the third cycle at 60 mm, the bottom of the steel tube exhibited noticeable bulging, taking on a “lantern” shape. During the first cycle at −72 mm, the concrete-filled steel tube specimen showed significant inclination, the horizontal load dropped sharply, and the test was stopped.

**Figure 10:**
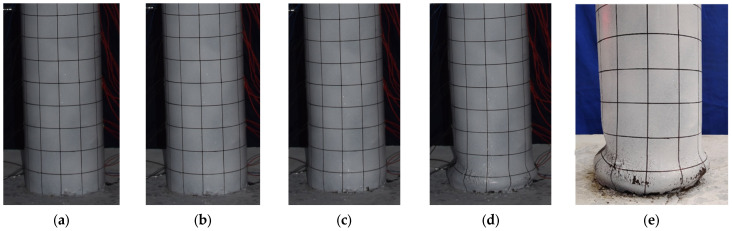
Damage development of specimen G-30. (**a**) ∆ = 18 mm; (**b**) ∆ = 36 mm; (**c**) ∆ = 48 mm; (**d**) ∆ = 60 mm; (**e**) final failure.

The damage development of specimen G-30 is shown in Figure 10. Slight separation between the steel tube and the concrete base was observed when the horizontal load reached 35 kN. During the third cycle at −18 mm, slight inward buckling of the steel tube on the void side was observed. During the third cycle at −36 mm, noticeable inward buckling deformation occurred in the steel tube on the left-gapped side within the range of 100 mm to 150 mm above the reinforced concrete base. During the third cycle at 48 mm, the bottom of the steel tube exhibited noticeable bulging, taking on a “lantern” shape. During the third cycle at 54 mm, the load on the pushing side dropped to 86% of the peak load, and the load on the pulling side (gapped side) dropped to 80% of the peak load, with significant inward buckling deformation occurring within the range of 50 mm to 100 mm above the reinforced concrete base on the gapped side. During the third cycle at −66 mm, the inward buckling deformation on the void side further increased, the bearing capacity dropped to 60%, and the test was stopped.

These four specimens exhibited similar failure modes and characteristics, but there were differences in the details. All specimens showed slight separation between the bottom of the steel tube and the reinforced concrete base when loaded to a certain lateral force. As the displacement increased, specimens G-0, G-10, and G-20 exhibited slight bulging at the bottom of the steel tube when the displacement reached between 36 mm and 48 mm. However, during the loading process, specimen G-30 showed noticeable inward buckling deformation on the gapped side at lower displacements (18 mm to 36 mm), indicating its relatively lower resistance to lateral displacement. After reaching the peak load, all four specimens exhibited a decrease in bearing capacity, especially as the displacement increased further. Specimens G-0, G-10, and G-20 showed noticeable bulging at the bottom of the steel tube when the displacement reached 60 mm. In contrast, specimen G-30 exhibited significant inward buckling deformation and a marked decrease in bearing capacity at a displacement of −66 mm, indicating an earlier failure.

### 3.2. Hysteresis Loop

**Figure 11:**
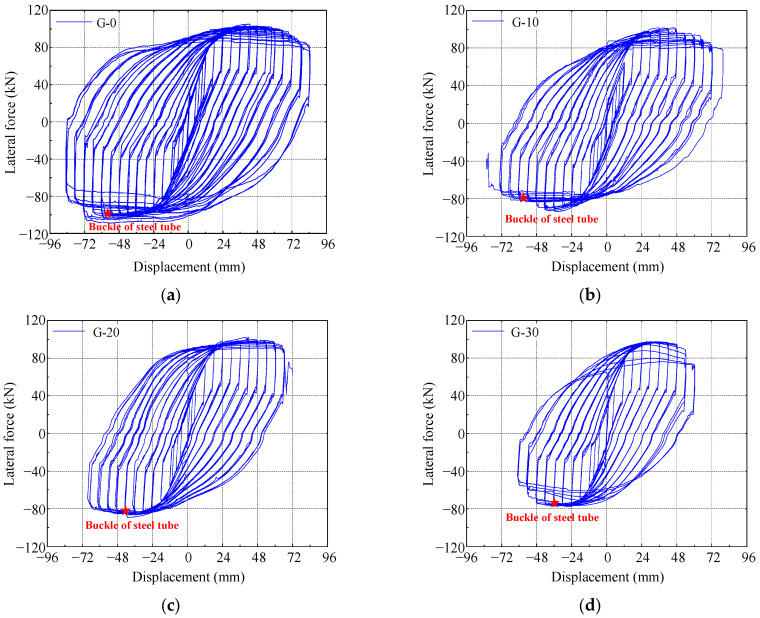
Lateral force–displacement hysteresis hoops. (**a**) G-0; (**b**) G-10; (**c**) G-20; (**d**) G-30.

**Figure 12:**
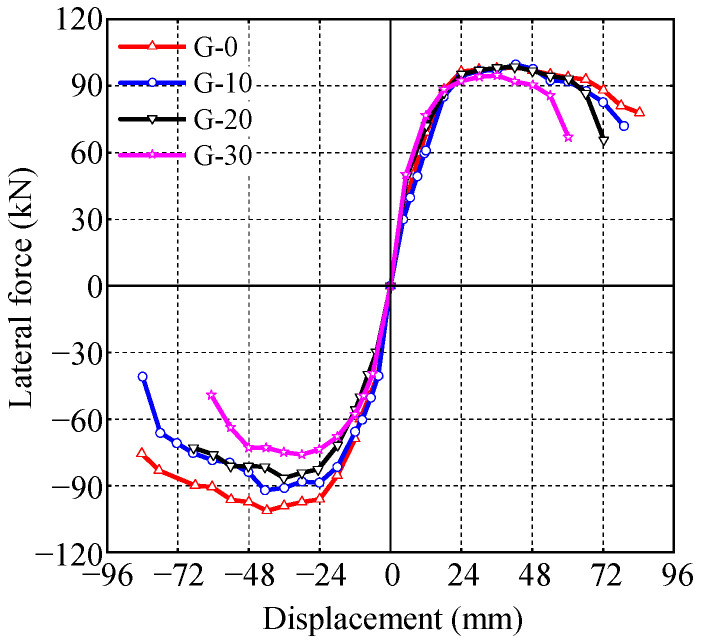
Skeleton curves of CFST specimens.

Lateral force–displacement hysteresis hoops of the specimens are shown in Figure 11. It should be noted that the lateral force and displacement is the response of the column under the combined action of vertical and lateral loads. In other words, the lateral force–displacement hysteresis loops include the P-Delta effect. The hysteresis loops of specimen G-0, which has no gap, are fuller compared to the other three specimens with gaps. In the initial loading phase, the specimens are in an elastic state, with small residual deformation after unloading. As the lateral displacement increases, bulging occurs at the bottom of the steel tube, and as the core concrete is crushed, the specimens enter a plastic state. During this phase, the residual deformation after unloading is larger, the hysteresis loops become fuller, and the energy dissipation of the specimens increases. After reaching the peak load, the lateral load gradually decreases with increasing displacement, and the area of the hysteresis loops also gradually decreases during the same level of loading. Subsequently, noticeable buckling of the steel tube occurs, and the hysteresis curve shows a clear decline, as marked by the pentagram in Figure 12.

### 3.3. Skeleton Curve, Bearing Capacity and Ductility

The envelope of the hysteresis loops is taken as the skeleton curve of the specimens, as shown in Figure 12. Based on the negative skeleton curves, the yield load and yield displacement of the specimens are calculated, using the equivalent energy method [31]. The characteristic points of the skeleton curve are listed in Table 4, where *F*_y_ and ∆_y_ represent the yield load and yield displacement, *F*_p_ and ∆_p_ represent the peak load and its corresponding displacement, and *F*_u_ and ∆_u_ represent the ultimate load and its corresponding displacement. The displacement ductility coefficient *μ* is calculated as follows:

(1) $$\mu =\frac{\Delta_{u}}{\Delta_{y}}$$

**Figure 13:**
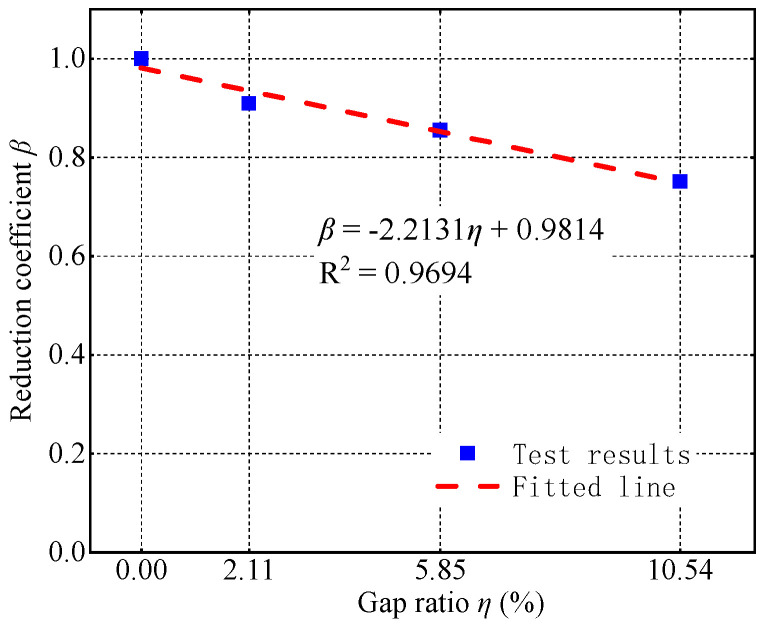
Bearing capacity reduction coefficient–gap ratio relationship.

The gap reduces the lateral load-bearing capacity of CFST columns. As the gap’s depth increases from 0 to 10 mm, 20 mm, and 30 mm, the negative bearing capacity decreases from 101.20 kN to 92.06 kN, 86.54 kN, and 76.04 kN, representing decreases of 9.03%, 14.49%, and 24.86%. The decrease in the negative bearing capacity could be attributed to two reasons. First, under negative loading, the side with gaps is subjected to compression, which reduces the effective compression area of the concrete and ultimately lowers the negative bearing capacity. Second, the presence of gaps weakens the steel tube’s confinement of the core concrete, resulting in a lower strength of the confined concrete compared to that without gaps, which, in turn, decreases the negative bearing capacity. To quantify the impact of gap ratio on hysteretic bearing capacity, a bearing capacity reduction coefficient *β* is defined:

(2) $$\beta =\frac{P_{\mathrm{gap}}}{P_{0}}$$

where $P_{\mathrm{gap}}$ denotes the hysteretic bearing capacity of the CFST column with gaps, and $P_{0}$ denotes the hysteretic bearing capacity of a CFST column without gaps. As shown in Figure 13, there is a clear linear relationship between the reduction coefficient *β* and the gap ratio *η*. So, the formula for calculating the reduction coefficient *β* of bearing capacity based on the gap ratio *η* was regressed:

(3) β=−2.2131η+0.9814

In practical engineering, if gaps are detected in a CFST column, its hysteretic bearing capacity must be reduced to assess the safety of the structure. For the convenience of designers, a table of hysteretic bearing capacity reduction coefficients is provided based on the test results, as shown in Table 5.

The existence of a gap leads to the specimens yielding under relatively low lateral forces, resulting in a decrease in their bearing capacity and ductility. As the gap’s depth increases from 0 to 10 mm, 20 mm, and 30 mm, the yield load of the specimens decreases from 88.32 kN to 79.12 kN, 75.65 kN, and 65.05 kN, respectively, representing decreases of 10.42%, 14.35%, and 26.35%. Similarly, the displacement ductility coefficient decreases from 4.214 to 3.552, 3.206, and 3.298, representing decreases of 15.7%, 23.9%, and 21.7%, respectively, as the gap’s depth increases from 0 to 10 mm, 20 mm, and 30 mm.

### 3.4. Energy Dissipation

**Figure 14:**
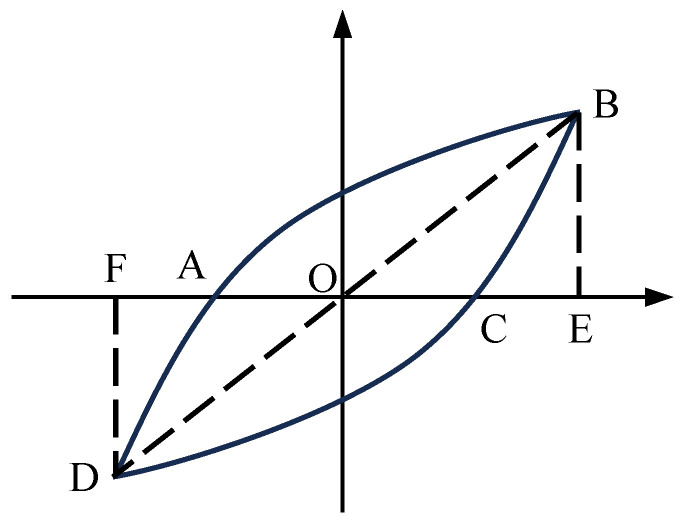
Definition of quantitative indicators of energy consumption capacity.

**Figure 15:**
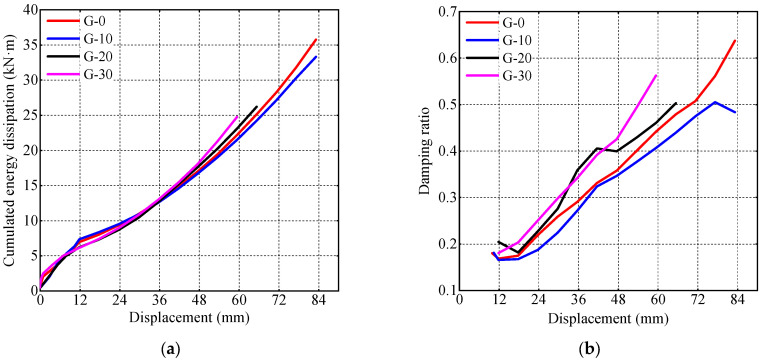
Energy dissipation of CFST specimens. (**a**) Cumulated energy dissipation; (**b**) damping ratio.

To quantify the energy dissipation capacity of the specimens, cumulative energy dissipation and equivalent viscous damping ratio *ξ*_eq_ are calculated according to Equation (4), where $S_{\mathrm{ABCD}}$ represents the area enclosed by the curve, with △OBE and △ODF denoting the areas under the curves, as illustrated in Figure 14. The cumulative energy dissipation and equivalent viscous damping ratio of the specimens at various displacements are shown in Figure 15.

The influence of gaps on the energy dissipation capacity of the specimens is minimal. As the depth of the gap increases, during the later stages of loading (lateral displacement greater than 48 mm), greater local buckling deformation occurs at the bottom of the steel tube. This results in earlier crushing of the core concrete, which absorbs energy. Consequently, specimens G-20 and G-30 exhibit slightly greater cumulative energy dissipation at the same horizontal displacement compared to specimens G-0 and G-10. Similarly, their viscous damping coefficient is also slightly higher than that of specimens G-0 and G-10.

(4) $$\xi_{\mathrm{eq}}=\frac{1}{2\pi }\frac{S_{\mathrm{ABCD}}}{S_{\mathrm{OBE}}+S_{\mathrm{ODF}}}$$

### 3.5. Stiffness Degradation

**Figure 16:**
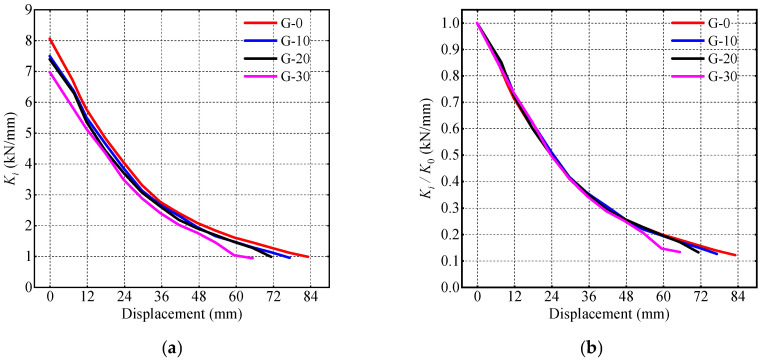
Stiffness degradation. (**a**) Secant stiffness; (**b**) stiffness ratio.

Stiffness is calculated as shown in Equation (5). Here, $K_{i}$ denotes the secant stiffness at the *i*-th load level, $\left|+P_{i}\right|$ and $\left|+\Delta_{i}\right|$ represent the positive peak load and corresponding displacement of the first hysteresis loop at the *i*-th load level, and $\left|-P_{i}\right|$ and $\left|-\Delta_{i}\right|$ represent the negative peak load and corresponding displacement of the first hysteresis loop at the *i*-th load level. Figure 16a illustrates the relationship between the specimen’s stiffness $K_{i}$ and the lateral displacement, and Figure 16b shows the relationship between the ratio of stiffness $K_{i}$ to initial stiffness $K_{i}$ and the lateral displacement. It can be observed that larger gap depths correspond to lower specimen stiffness. However, the stiffness ratios of all specimens overlap before reaching a lateral displacement of 48 mm. As the displacement exceeded 48 mm, specimen G-30’s steel tube exhibited significant buckling, resulting in a rapid decrease in stiffness that is notably lower than the other specimens’.

(5) $$K_{i}=\frac{\left|+P_{i}\right|+\left|-P_{i}\right|}{\left|+\Delta_{i}\right|+\left|-\Delta_{i}\right|}$$

### 3.6. Strain of Steel Tube

**Figure 17:**
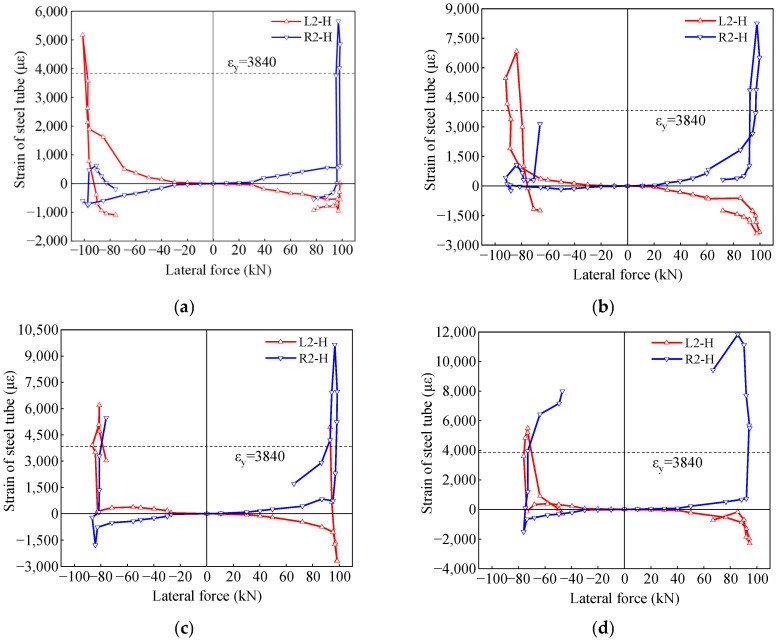
Strain in the hoop direction. (**a**) G-0; (**b**) G-10; (**c**) G-20; (**d**) G-30.

The strain of the steel tube in the circumferential direction at a height of 150 mm from the top surface of the base is depicted in Figure 17. Due to the buckling deformation at the bottom of the steel tube, the circumferential strains L2-H and R2-H at the bottom primarily exhibit tensile strains. After reaching peak load, the circumferential strains suddenly decreased. The peak strain in L2-H is smaller than the peak strain in R2-H because the steel tube at R2-H was compressed by the expansion of the core concrete, and the circumferential strain of the steel tube at this location increases. The presence of the gap at the bottom left reduced the expansion and compressing effect of the core concrete on the steel tube at L2-H.

Observing the circumferential strain (L2-H) of the steel tube on the gap side during compression, represented by the red line under negative loading in Figure 17, it was found that for specimens G-0 and G-10, the circumferential strain of the steel tube gradually increased with the increase in lateral force until the peak load. However, for specimens G-20 and G-30, the circumferential strain of the steel tube initially increased with the increase in lateral force, but then began to gradually decrease when the lateral force was between 60 kN and the peak load. This indicates that the confinement effect of the steel tube on the concrete was gradually decreasing. This may be due to the lack of support from the core concrete on the gap side of the steel tube, resulting in the initiation of inward buckling of the steel tube.

## 4. Conclusions

This study conducted quasi-static tests on four concrete-filled circular steel specimens with varying depths of spherical-cap gap to investigate their hysteretic performance. The analysis focused on the specimens’ load-bearing capacity, ductility, cumulative energy dissipation, stiffness degradation, and steel tube strains. The main conclusions are as follows:

1. The load-bearing capacity and ductility of the concrete-filled steel specimens gradually decreased with increasing gap depth. Specifically, as the gap’s depth increased from 0 mm to 10 mm, 20 mm, and 30 mm, the load-bearing capacity decreased by 9.03%, 14.49%, and 24.86%, respectively, and ductility also decreased by 15.7%, 23.9%, and 21.7% correspondingly.
2. A reduction coefficient *β* was proposed to account for the influence of spherical-cap gaps on the seismic bearing capacity of CFST columns. Based on the experimental results, a calculation method for *β* based on the gap ratio was established, providing a reference for the assessment of the bearing capacity of CFST structures with spherical-cap gaps.
3. Spherical-cap gaps reduced the cumulative energy dissipation and initial stiffness of the concrete-filled steel tubes. The initial stiffness of the concrete-filled steel tubes decreased with the increase in gap depths, although the impact of gaps on the rate of stiffness degradation was little.
4. Spherical-cap gaps diminished the confinement effect of the steel tube on the core concrete, causing premature yielding of the steel tube on the gap side. Since the gap weakened the compressing effect on the steel tube exerted by the expansion of core concrete, the peak circumferential strain of the steel tube at the gap side was smaller than that of the steel tube at the non-gap side.

In this paper, the influence of spherical-cap gaps on the hysteretic behavior of CFST columns was quantified through quasi-static experiments. The conclusion is based on the experimental results, which have certain limitations. Numerical simulations are highly necessary for future research in this area, as it is vital to assess seismic performance at the scale of actual buildings.

## Figures and Tables

**Table 1:** Parameters of specimens (unit: mm).

Specimen	Diameter	Thickness of Steel Tube	Gap Depth	Gap Ratio *η*	Specimen Height
G-0	180	6	0	0	1200
G-10	180	6	10	2.11%	1200
G-20	180	6	20	5.85%	1200
G-30	180	6	30	10.54%	1200

Notes: gap ratio represents the ratio of the gap area to the total cross-sectional area.

**Table 2:** Material properties of concrete.

Item	1	2	3	Mean Value
Compressive strength of cube (MPa)	50.5	46.8	54.0	50.4
Compressive strength of prismatic (MPa)	30.6	26.1	31.1	29.3
Elastic module (MPa)	30,961	32,747	31,679	31,796

**Table 3:** Material properties of steel tube.

Item	Yield Strength *f*_y_ (mpa)	Yield Strain *ε*_y_ (*ε*)	Ultimate Strength *f*_u_ (mpa)	Ultimate Strain *ε*_u_ (*ε*)	Elastic Module *E*_s_ (mpa)
Mean value	378.58	0.00384	553.75	0.171	206,434

**Table 4:** Characteristic points of the specimens.

Specimen	*F*_y_ (kN)	∆_y_ (mm)	*F*_p_ (kN)	∆_p_ (mm)	*F*_u_ (kN)	∆_u_ (mm)	*μ*	Ductility Reduction Ratio
G-0	88.32	18.05	101.20	41.89	83.60	76.06	4.214	/
G-10	79.12	17.11	92.06	42.6	78.25	60.78	3.552	15.7%
G-20	75.65	20.35	86.54	36.07	73.56	65.24	3.206	23.9%
G-30	65.05	16.25	76.04	30.05	64.64	53.60	3.298	21.7%

**Table 5:** Reduction coefficient for bearing capacity.

Gap Ratio	2.11%	5.85%	10.54%
Reduction coefficient *β*	0.91	0.86	0.75

## Data Availability

Data are contained within the article.
